# A Randomized Clinical Trial Testing the Anti-Inflammatory Effects of Preemptive Inhaled Nitric Oxide in Human Liver Transplantation

**DOI:** 10.1371/journal.pone.0086053

**Published:** 2014-02-12

**Authors:** John D. Lang, Alvin B. Smith, Angela Brandon, Kelley M. Bradley, Yuliang Liu, Wei Li, D. Ralph Crowe, Nirag C. Jhala, Richard C. Cross, Luc Frenette, Kenneth Martay, Youri L. Vater, Alexander A. Vitin, Gregory A. Dembo, Derek A. DuBay, J. Steven Bynon, Jeff M. Szychowski, Jorge D. Reyes, Jeffrey B. Halldorson, Stephen C. Rayhill, Andre A. Dick, Ramasamy Bakthavatsalam, Jared Brandenberger, Jo Ann Broeckel-Elrod, Laura Sissons-Ross, Terry Jordan, Lucinda Y. Chen, Arunotai Siriussawakul, Devin E. Eckhoff, Rakesh P. Patel

**Affiliations:** 1 Department of Anesthesiology and Pain Medicine, University of Washington School of Medicine, Seattle, Washington, United States of America; 2 Department of Surgery, University of Washington School of Medicine, Seattle, Washington, United States of America; 3 Department of Hepatobiliary-pancreatic Surgery, China-Japan Union Hospital of Jilin University, Changchun, China; 4 Department of Anesthesiology, University of Alabama at Birmingham, Birmingham, Alabama, United States of America; 5 Department of Pathology, University of Alabama at Birmingham, Birmingham, Alabama, United States of America; 6 Department of Biostatistics, University of Alabama at Birmingham, Birmingham, Alabama, United States of America; 7 Department of Surgery, University of Alabama at Birmingham, Birmingham, Alabama, United States of America; 8 Department of Pathology and Laboratory Medicine, Ruth and Raymond Perelman School of Medicine, Philadelphia, Pennsylvania, United States of America; 9 Department of Surgery, Division of Immunology and Organ Transplantation, University of Texas Health Science Center at Houston, Houston, Texas, United States of America; 10 Department of Surgery, University of California San Diego Health Care System, San Diego, California, United States of America; 11 Department of Anesthesiology, Faculty of Medicine, Siriraj Hospital, Mahidol University, Bangkok, Thailand; University of California Los Angeles, United States of America

## Abstract

Decreases in endothelial nitric oxide synthase derived nitric oxide (NO) production during liver transplantation promotes injury. We hypothesized that preemptive inhaled NO (iNO) would improve allograft function (primary) and reduce complications post-transplantation (secondary). Patients at two university centers (Center A and B) were randomized to receive placebo (n = 20/center) or iNO (80 ppm, n = 20/center) during the operative phase of liver transplantation. Data were analyzed at set intervals for up to 9-months post-transplantation and compared between groups. Patient characteristics and outcomes were examined with the Mann-Whitney U test, Student t-test, logistic regression, repeated measures ANOVA, and Cox proportional hazards models. Combined and site stratified analyses were performed. MELD scores were significantly higher at Center B (22.5 vs. 19.5, p<0.0001), surgical times were greater at Center B (7.7 vs. 4.5 hrs, p<0.001) and warm ischemia times were greater at Center B (95.4 vs. 69.7 min, p<0.0001). No adverse metabolic or hematologic effects from iNO occurred. iNO enhanced allograft function indexed by liver function tests (Center B, p<0.05; and p<0.03 for ALT with center data combined) and reduced complications at 9-months (Center A and B, p = 0.0062, OR = 0.15, 95% CI (0.04, 0.59)). ICU (p = 0.47) and hospital length of stay (p = 0.49) were not decreased. iNO increased concentrations of nitrate (p<0.001), nitrite (p<0.001) and nitrosylhemoglobin (p<0.001), with nitrite being postulated as a protective mechanism. Mean costs of iNO were $1,020 per transplant. iNO was safe and improved allograft function at one center and trended toward improving allograft function at the other. ClinicalTrials.gov with registry number 00582010 and the following URL:http://clinicaltrials.gov/show/NCT00582010.

## Introduction

Liver transplantation is the only curative treatment for acute and chronic liver failure. Though life-saving, liver transplantation is one of the most expensive and resource-intensive interventions that contemporary medicine has to offer, highlighting the need for additional therapies to assist in reducing costs and care intensity [1]. Moreover, the need for liver transplantation continues to escalate in the United States with the number of transplants estimated at ∼6,500 per year, a demand far exceeding current capabilities [2]. While improvements in perioperative management and surgical technique have led to improved morbidity and mortality, acute or chronic graft dysfunction attributable to ischemia-reperfusion injury (IRI) remains significant (∼30%) [3]. In fact, hepatic IRI occurs during all liver transplants and is associated with primary graft non-function, delayed graft function and other complications resulting in greater intensity of care, prolonged hospital length of stay or increased hospital readmissions, thus greater costs.

IRI in the liver results in disturbances of the sinusoidal microcirculation and the generation of various pro-inflammatory mediators such as reactive oxygen species (ROS), cytokines, activation of chemokines and other cell signaling molecules [4]. In addition, decreased nitric oxide (NO) bioavailability, secondary to either decreased production from endothelial nitric oxide synthase (eNOS) and/or diversion of NO away from physiologic signaling targets by reactions with superoxide anion radical, lipid radicals, or metallo/heme proteins, has been noted during liver IRI [5], [6], [7]. The consequences of decreased NO bioavailability include increased oxidative stress, increased apoptosis, increased leukocyte adhesion, reduced microcirculatory flow, and perturbed mitochondrial function [6], [8]. Importantly, therapeutic restoration of NO leads to protection against liver IRI underscoring the importance of NO-signaling as a mediator and therapeutic target for IRI during liver transplantation [9],[10], [11], [12].

Administration of iNO has been touted as therapy for numerous cardiopulmonary disorders. Unfortunately, its use in adults has met with limited success with the prospective clinical trials and other clinical evidence not favoring it as a first line therapy in these patients [13]. Conventional reasoning has been that as iNO crosses the alveolar-capillary membrane it rapidly reacts with oxy- or deoxy hemoglobin in the red blood cell (RBC) and is rendered inactive. However, seminal studies by Fox-Robichaud et al dismissed this concept, demonstrating that iNO exhibits extra-pulmonary bioactivity in the mesenteric vasculature by preventing neutrophil adhesion in a feline model of IRI [14]. These concepts have been extended in other studies demonstrating attenuation in organ inflammation and injury [10], [11], [15], [16], [17], [18]. Our previous study performed at one center, demonstrated that iNO therapy (80ppm administered during the intraoperative period) attenuated IRI and improved liver function post-transplantation. In the current study we employed a prospective, blinded randomized placebo-controlled trial design conducted at two established large volume university liver transplant centers, to test the effectiveness of iNO therapy for enhancing allograft function in the immediate post-operative period and reducing longer term complications.

## Results

### Patients and surgery demographics

**Figure 1:**
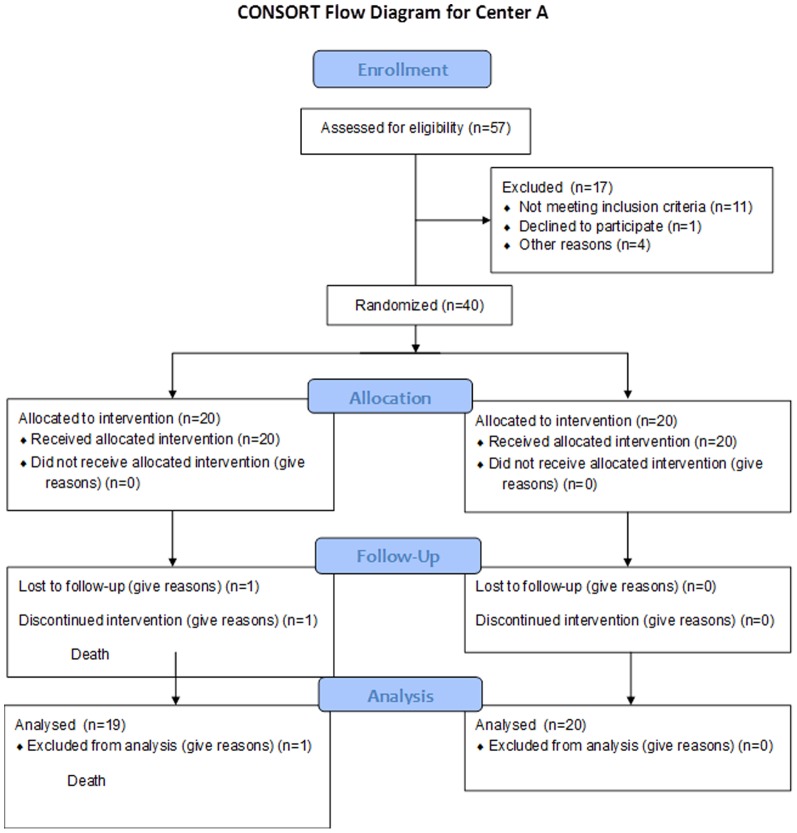
CONSORT diagram for center A. Assessment of Eligibility and Inclusion in the Inhaled Nitric Oxide for Attenuating Liver Allograft Injury Study at Center A.

**Figure 2:**
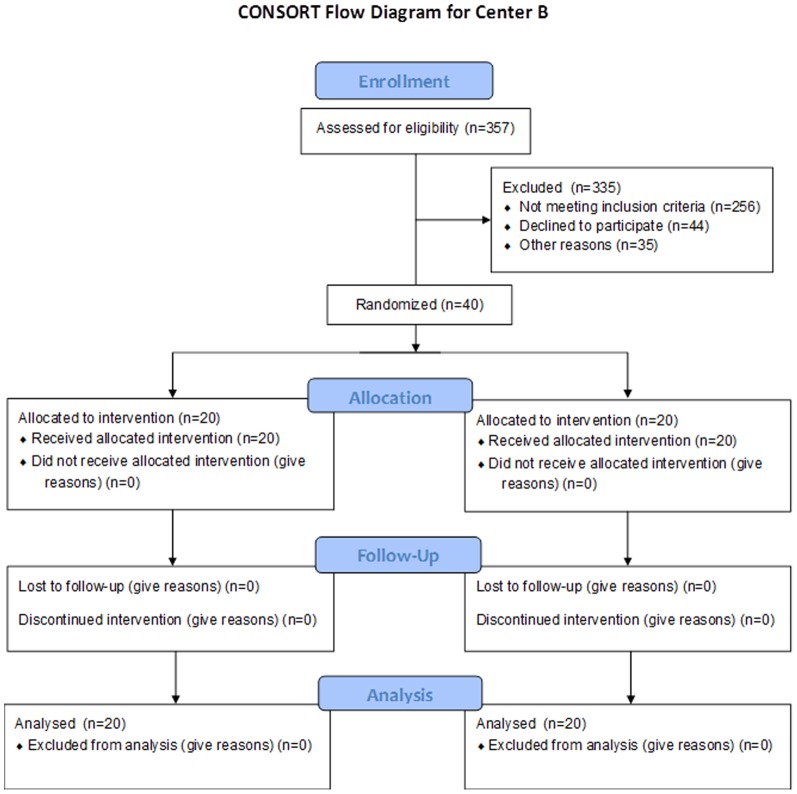
CONSORT diagram for center B. Assessment of Eligibility and Inclusion in the Inhaled Nitric Oxide for Attenuating Liver Allograft Injury Study at Center B.

**Figure 3:**
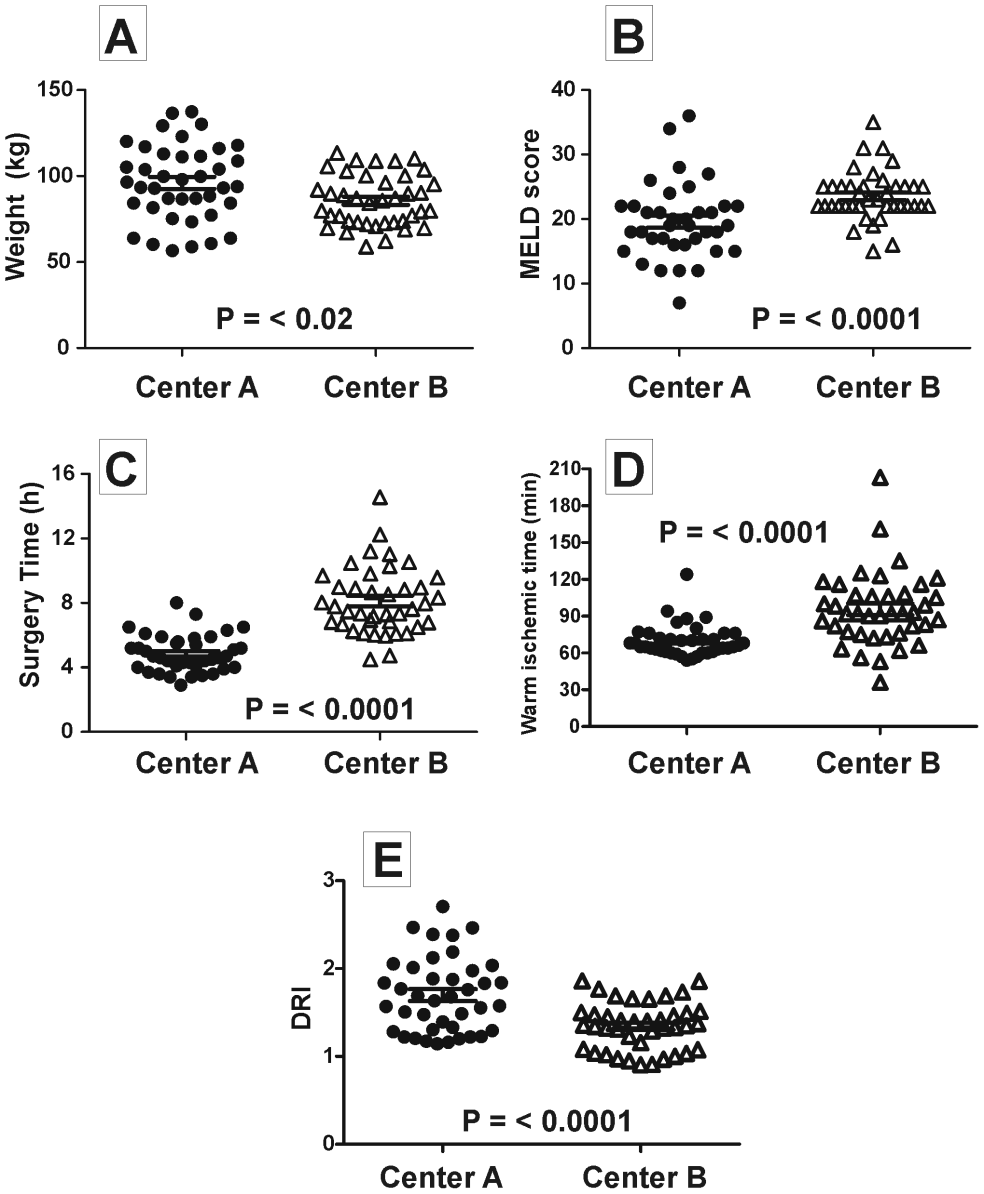
Patient demographic differences between study sites. Panels A–E show respectively patient weight, MELD scores, surgery, warm ischemic times and donor risk index (DRI). Each data point represents individual patient (n = 37–40 for Center A, and n = 40 for Center B). Indicated P-values are from unpaired t-test (panel A and E) or Mann-Whitney U test (panels B–D).

Demographics for Center A and Center B cohorts according to CONSORT guidelines are reported (Table 1 and 2, and Figure 1 and 2). Within each site, no differences in recipient or donor age, weight, MELD scores, DRI, cold ischemic time for donor liver, time of surgery or time between portal vein unclamp and hepatic artery unclamp (min) was observed between placebo and iNO groups. Significant differences between sites in patient weight (higher at Center A vs. Center B, Fig. 3A; BMI (in kg/m^2^) were 30.9±1.12 and 28.7±0.66 (mean ± SEM for center A and B respectively, P = 0.086)), MELD scores (lower at Center A vs. Center B, Figure 3B), surgery times (shorter at Center A vs. Center B, Figure 3C), WIT (lower at Center A vs. Center B, Figure 3D) and DRI (higher at Center A vs. Center B, Figure 3E) were observed. Since these baseline differences may affect the severity of IRI and possible responsiveness to iNO [19], we present data stratified by center but combined in specific cases.

**Table 1:** Patient and Surgery Demographics for Center A Cohort.

Center A	Placebo (*n* = 20)	iNO (*n* = 20)	*P*
Age (yr)	55 (45–68)	54 (36–72)	0.59
Sex	16 males, 4 females	16 males, 4 females	>0.99
Weight (kg)	98 (57–137)	94 (59–137)	0.69
Race	19 Caucasian, 1 African American	20 Caucasian	>0.99
Lab-MELD	19 (12–25)	20 (7–36)	0.31
ASA classification (no.)	4 (17), 3 (3)	4 (18), 3 (2)	>0.99
Cold ischemic time (min)	493.5 (240–749)^B^	472.5 (155–760)	0.94
Total surgical time (h)	4.5 (3.4–6.5)^A^	4.6 (3.5–7.3)	0.81
Total Warm Ischemic Time (min)	68 (57–124)^A^	64.5 (54–94)	0.3
Preservation solution	UW (16), SPS1 (4)	UW (15), SPS 1 (5)	>0.99
Donor age (yr)	47 (14–74)	50 (13–74)	0.69
Indication for	Hepatitis C (4) Alcoholic cirrhosis (3)	Hepatitis C (4) Hepatitis C/Cirrhosis (2)	N/A
transplantation (no.)	Primary biliary cirrhosis (3)	Hepatocellular carcinoma/Nonalcoholic	
	Hepatitis C/Cirrhosis (2)	steatohepatitis (2)	
	Nonalcoholic steatohepatitis (2)	Hepatitis C/Primary biliary cirrhosis (2)	
	Sclerosing cholangitis (2)	Nonalcoholic steatohepatitis (1)	
	Laennec's Cirrhosis (1)	Hepatocellular carcinoma (1)	
	Hepatic Hydrothorax/Cirrhosis (1)	Cryptogenic cirrhosis(1)	
	Cirrhosis (2)	Hepatitis C/Alcoholic cirrhosis (1)	
		Fatty Liver (1)	
		Sclerosing cholangitis (1)	
		Cholelithiasis/ESLD (1)	
		Alcoholic cirrhosis (1)	
Postoperative	Renal dysfunction (5) Edema (4)	Edema (4) Anemia (3)	N/A
complications (no.)	Allograft dysfunction (3) Anemia (3)	Hyperglycemia (3)	
	Cholestasis (3) Neuropathy (3)	Pleural effusion (2)	
	Pleural effusion (3)	Renal dysfunction (2)	
	Hyperglycemia (2)	Hyperkalemia (2) Neuropathy (2)	
	Tachycardia (1)	Allograft dysfunction (1)	
	Hepatocellular carcinoma (2)	Tachycardia (1) Hypertension (1)	
	Pulmonary edema (1)	Hepatocellular carcinoma (1)	
	Pneumonia (1) Hypervolemia (1)	Pneumonia (1) Pulmonary	
	Atrial fibrillation (1)	Hypertension (1) Wound abscess (1)	
	Occlusive thrombus (1)	Atrial fibrillation (1) Thrombus (1)	
	Cryptococcal meningitis (1)	Weight loss (1)	
	Bacteremia (1) Dysphasia (1)	Infected hematoma (1)	
	Hypomagnesemia (1), Weight loss (1)	Hepatic abscess (1)	
	Adhesions/small bowel obstruction (1)		
	Biliary cast syndrome (1)		
	Sclerosing cholangitis (1)		
	Post-cardiac arrest syndrome		
	(intraoperative cardiac arrest)		
	Re-transplant (1)		

Values show median (range). P-values calculated from unpaired t-test for placebo vs. iNO. N = 20 except An = 19. Bn = 18.

**Table 2:** Patient and Surgery Demographics for Center B Cohort.

UW	Placebo (*n* = 20)	iNO (*n* = 20)	*P*
Age (yr)	57 (24–70)	59 (30–67)	0.88
Sex	17 Males, 3 Females	15 Males, 5 Females	0.69
Weight (kg)	87 (59–113)	82(62–109)	0.80
Race	15 Caucasian, 1 African American, 4 Other	17 Caucasian, 1 African American, 2 Other	0.83
Lab-MELD	23 (18–35)	22 (15–31)	0.42
ASA classification (no.)	3 (11), 3E (4), 4 (4), 4E(1)	3 (13), 3E (1), 4 (4), 4E (2)	0.61
Cold ischemic time (min)	529 (277–1043)	518 (220–794)	0.54
Total surgical time (h)	7.9 (4.5–12.2)	7.5 (4.7–14.6)	0.96
Warm Ischemic Time (min)	92.5 (53–203)	91 (36–161)	0.57
Preservation solution	UW (16), HKT (4), SPS1 (1)	UW (18), HKT (2)	0.53
Donor age (yr)	37.5 (17–58)	46 (20–67)	0.37
Indication for	Hepatitis C/Hepatocellular carcinoma (10)	Hepatitis C/Hepatocellular	N/A
transplantation (no.)	Hepatitis C (3)	carcinoma (9)	
	Hepatitis C/Alcoholic cirrhosis (2)	Hepatitis C/Alcoholic cirrhosis (3)	
	Primary biliary cirrhosis/Hepatocellular	Hepatitis C (2)	
	carcinoma (2)	Alcoholic cirrhosis (1)	
	Hepatitis B/Alcoholic cirrhosis (1)	Primary biliary cirrhosis (2)	
	Autoimmune hepatitis/cirrhosis (1)	Autoimmune hepatitis/Primary	
	Alcoholic cirrhosis (1)	biliary cirrhosis (1)	
		Cryptogenic cirrhosis (1)	
Postoperative	Tacrolimus toxicity (3) Rejection (3)	Tacrolimus toxicity (1)	N/A
complications (no.)	Malnutrition/failure to thrive (3)	Hepatic artery stenosis (1)	
	Incisional ventral hernia (3) Hyperglycemia (2)	Hypertension (1)	
	Anemia (2) Renal dysfunction (2)	Malnutrition/failure to thrive (1)	
	Hepatic artery stenosis (2)	Hypomagnesemia (1)	
	Biliary obstruction (2) Hypercalcemia (1)	Hyperkalemia (1) Hemothorax (1)	
	Hyperkalemia (1)	Hyperbilirubinema (1)	
	Hypovolemia (1) Hyperbilirubinemia (1)	Renal dysfunction (1)	
	Hypertension (1)	Pleural effusion (1)	
	Thrombocytopenia (1) Coagulopathy (1)	Diaphragmatic hemiparesis (1)	
	Pleural effusion (1)	Incisional ventral hernia (1)	
	Perihepatic fluid collection (1)	Cardiac arrest/death (1)	
	Hyperammonemia (1) Bile leak (1)		
	Respiratory failure (1) Hypervolemia (1)		
	Bilateral inguinal hernia (1)		
	Abdominal distension (1)		
	Re-transplant (1) Cardiac arrest/death (2)		

Values show median (range). P-values calculated from unpaired t-test for placebo vs. iNO.

### Assessment of iNO safety

**Figure 4:**
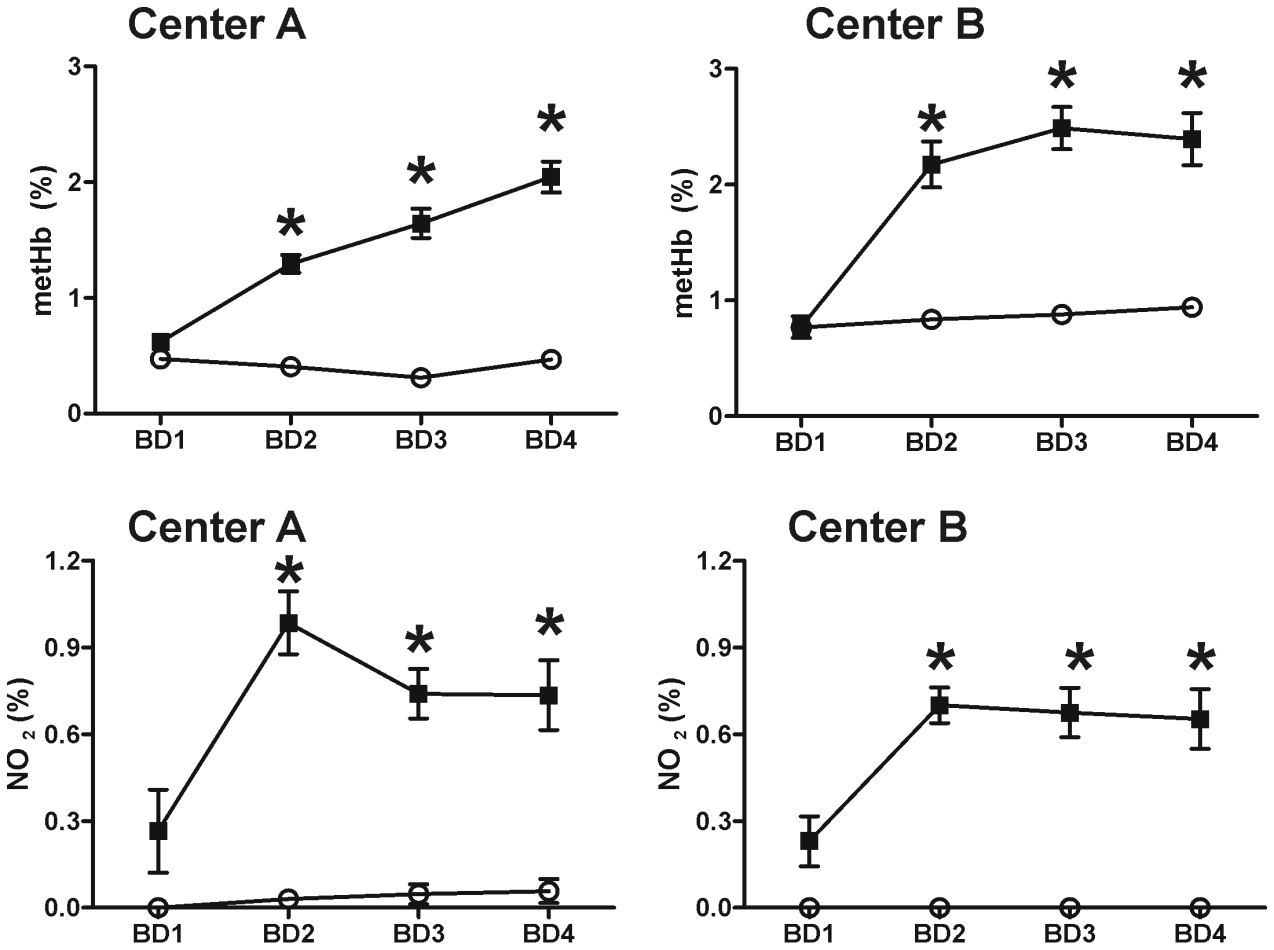
Changes in metHb and nitrogen dioxide (NO_2_) as a function of blood draw (BD) in placebo (○) or iNO (▪) groups. Data are mean ± SEM (n = 20 for each group, except UAB placebo where n = 19 and UW iNO where n = 17–20. *P<0.0001 by 2-way ANOVA with Bonferroni multiple comparisons post-test.

**Figure 5:**
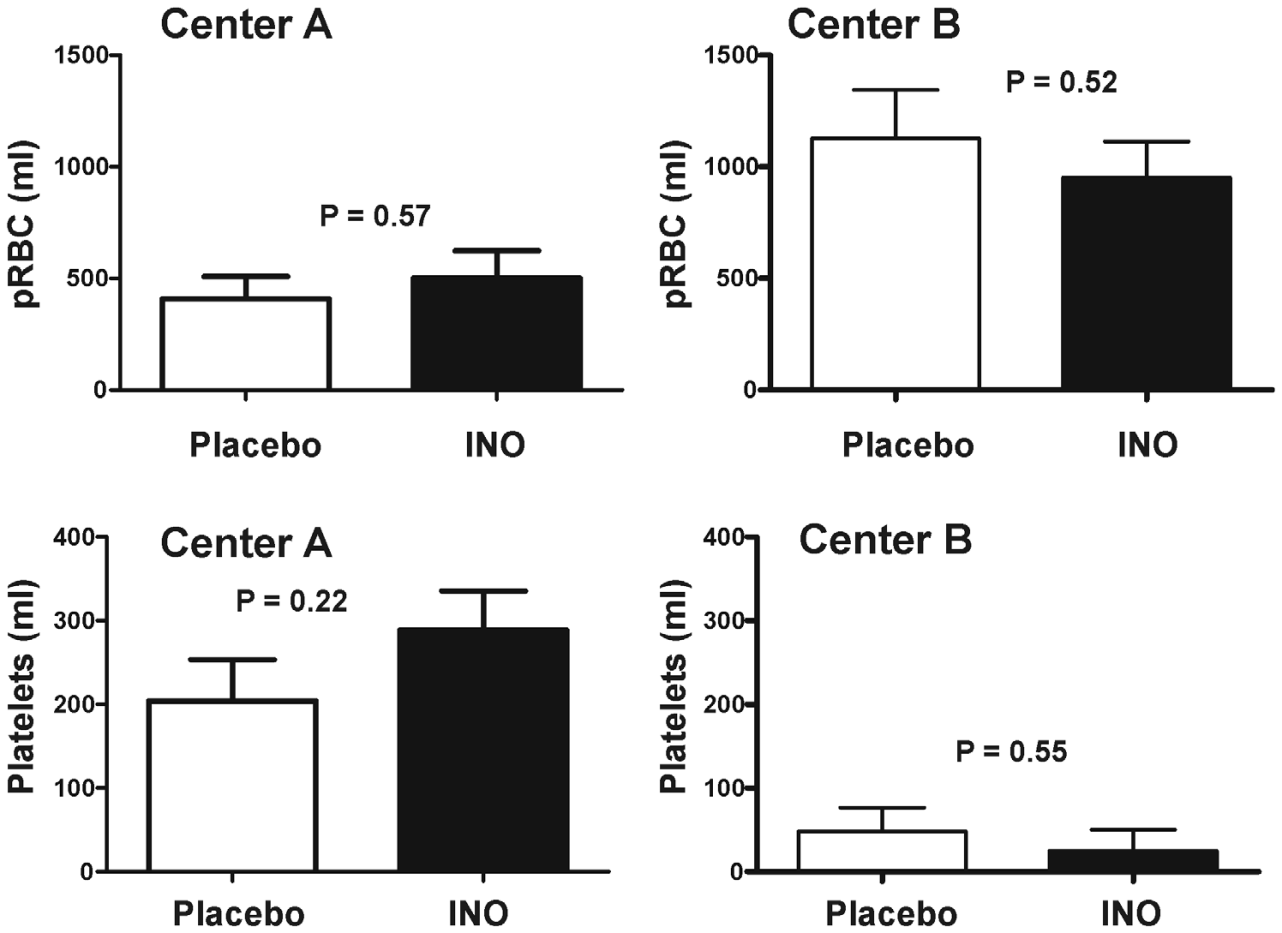
iNO therapy did not affect volume of packed RBC (pRBC) or platelets transfused during liver transplant surgery at either study site. Data are mean ± SEM (n = 20 for each group, except UAB placebo where n = 19). Indicated p-values calculated by unpaired t-test.

Consistent with our previous study, no adverse effects of iNO were observed [11]. Levels of nitrogen dioxide (NO_2_) radical and metHb increased in iNO groups relative to placebo but remained below safety threshold levels (Figure 4). No significant effects on platelet or RBC transfusion were observed between iNO and placebo within either center (Figure 5).

### Effects of iNO on post-transplantation liver function tests

**Figure 6:**
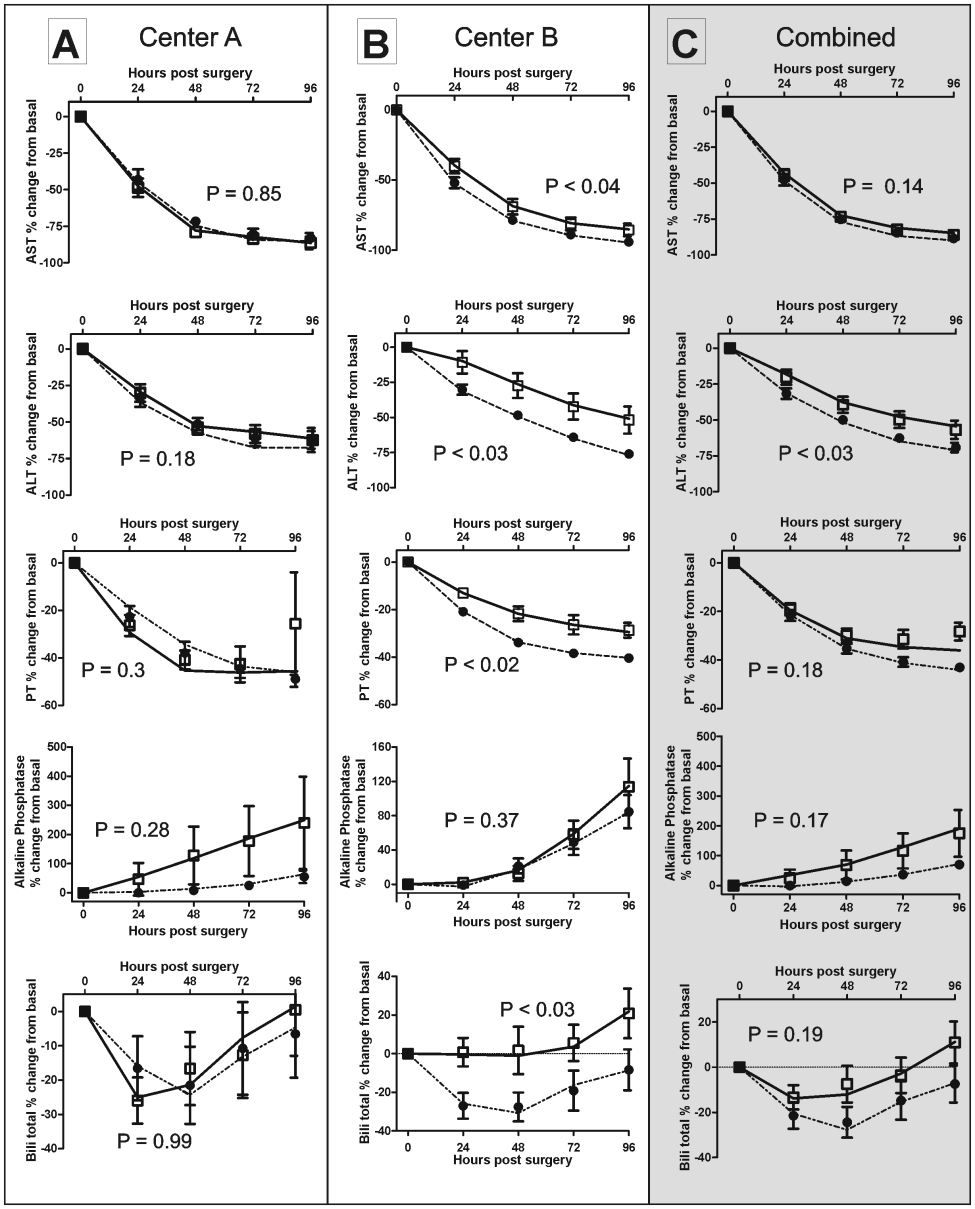
Center dependent effects of iNO therapy on post-transplant liver function. Liver function post-transplantation was assessed by following post-surgery time-dependent changes in serum AST, serum ALT, PT time, alkaline phosphatase and bilirubin as indicated. Panels A,B, C shows respectively data for Center A, Center B and the two centers combined. Data represented by symbols (□ = placebo, • = iNO) are presented as percent change relative to first measurement post-surgery and are mean ± SEM (n = 20, except Center A placebo where n = 19; n = 39–40 for combined). Fitted lines (solid line = placebo, dotted line = iNO) show means after adjustment for MELD, cold ischemic time, weight, BMI (>25), warm ischemia time (WIT) and donor age. P-values indicate significance of iNO vs. placebo for adjusted data. Absolute values for LFT parameters measured immediately (<1 h) post-surgery for were: AST 722.8±114 U/L (placebo) and 636.1±136.9 U/L (iNO) at Center A; 1432.1±222.5 U/L (placebo) and 1429.1±134.3 U/L (iNO) at Center B; ALT 479.5±67.1 U/L (placebo) and 423.7±77.8 U/L (iNO) at Center A; 904.3±155.4 U/L (placebo) and 987.7±109.2 U/L (iNO) at Center B; PT time 31.9±2.4 s (placebo) and 31.7±2 s (iNO) at Center A; 22.6±0.8 s (placebo) and 24.6±0.8 s (iNO) at Center B; Alk Phos 77.7±14.3 U/L (placebo) and 70.1±8.9 U/L (iNO) at Center A; 57.2±4.4 U/L (placebo) and 68.4±13 U/L (iNO) at Center B; Bili-total 3.6±0.4 (placebo) and 3.66±0.33 (iNO) at Center A; 3.2±0.5 (placebo) and 4.2±0.5 U/L (iNO) at Center B. Data are normalized to ALT and AST levels measured immediately (<1 h) post-surgery and were 601.8±145.4 U/L (placebo) and 689.3±149.5 U/L (iNO) for ALT and 922.1±228.7 U/L (placebo) and 940.9±211.3 U/L (iNO) for AST.

The average percent changes in AST, ALT, PT, AP and bilirubin were plotted as a function of time post-surgery relative to the first measurement taken (Figure 6). For all LFT outcomes, no significant interaction between the treatment and time after surgery was observed, indicating that any treatment effect is consistent across all post-surgery time points. The DRI is a significant factor in predicting outcomes after liver transplantation [20]. Since demographics for each center differed significantly (Figure 3), initial mixed model analysis using DRI and the center as covariates was performed and indicated that changes in AST were significantly different in iNO vs. placebo, with no treatment effect being noted for any of the other LFT except for PT which was only significantly different between iNO and placebo in the Center B cohort. Since the other demographic factors that differed between Center A and B, could influence IRI responses also we performed a mixed model analysis adjusting for MELD, weight, BMI (>25), cold ischemic time, surgery time, WIT, donor age for each center cohort and on combined cohorts (Figure 3). A significant treatment effect was observed for ALT for the entire cohort; levels decreasing more rapidly in iNO treated group vs. placebo. However, no treatment effect in the entire cohort was observed with AST, PT, alkaline phosphatase or bilirubin. Center specific analysis indicated however that PT, AST and bilirubin were significantly different between iNO and placebo only at Center B. Collectively, these data indicate that iNO therapy improves allograft function across both centers when indexed by ALT, a specific enzyme for liver injury and then broad based assessment using multiple LFT indicate a significant therapeutic effect of iNO only in Center B.

### Effects of iNO on SICU and hospital length of stay

**Figure 7:**
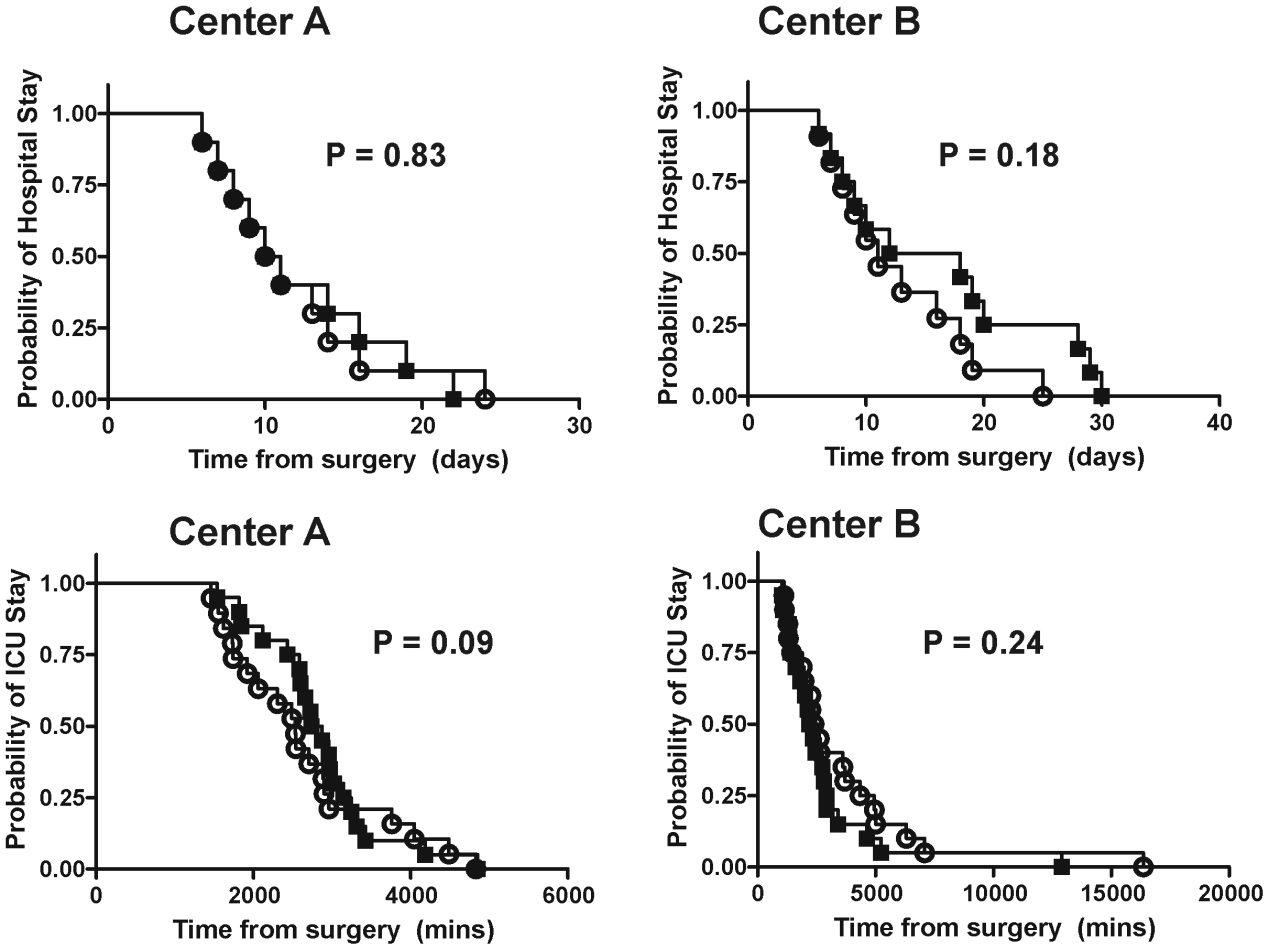
iNO therapy did not affect hospital or ICU length of stay. Kaplan-Meier plots shown and with P-values from Cox proportional hazards models adjusting for MELD, cold ischemic time, weight, BMI, warm ischemic time or donor age within each center. Placebo = ○, iNO = ▪.

iNO had no effect on SICU or hospital length of stay at either center (Table 3). Moreover, Kaplan-Meier analysis did not show any significant differences with or without Cox regression modeling and adjusting for MELD, cold ischemic time, weight, BMI, WIT or donor age within each center or when combined (Figure 7).

**Table 3:** Effect of iNO on Patient SICU and Hospital Length of Stay.

	Center A	Center A	p	Center B	Center B	p
Characteristics	Placebo	iNO		Placebo	iNO	
Hospital Stay (days)*	9 (7–13)	8.5 (8–12.5)	0.83	9 (7–12)	9.5 (7.5–18.5)	0.49
ICU Stay (min)*	2534 (1743–2963)	2808 (2469–3221	0.21	2503 (1663–4641)	2259 (1497–2930)	0.47

* median and interquartile range. P-values by Mann-Whitney unpaired t-test.

### Effects of iNO on post-operative hepatobiliary and all-cause complications

**Figure 8:**
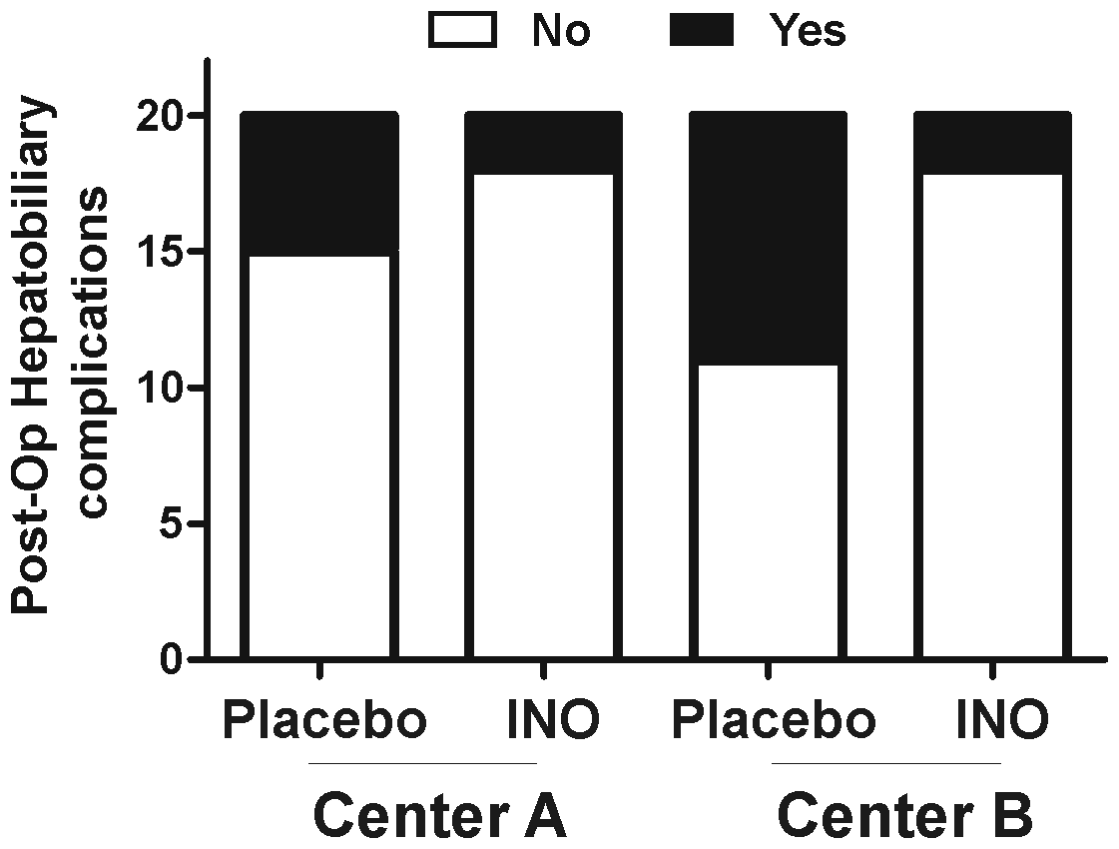
Rates of post-operative hepatobiliary complications. A. Data are presented as ‘no’ or ‘yes’ indicating the absence or presence of hepatobiliary complications respectively within 9 months of surgery.

In a post-hoc analysis, the number of post-operative hepatobiliary complications within the initial 9 months after transplantation were recorded (Figure 8 and Table 4). In the Center A cohort, complications were 10% vs. 25% in iNO and placebo groups, respectively. In the Center B cohort, complications were 10% vs. 45% in iNO and placebo groups, respectively. Logistic regression model analyses adjusted for MELD showed no significant change in the odds of complications between iNO and placebo groups at Center A (p = 0.13, OR = 0.17, 95% CI (0.02, 1.64), but significant differences were observed in the Center B cohort (p = 0.02, OR = 0.125, 95% CI (0.02, 0.72). Significance was maintained when centers were combined with hepatobiliary complications decreasing from 35% to 10% in placebo vs. iNO respectively (p = 0.0062, OR = 0.15, 95% CI (0.04, 0.59). Additionally, the number of all-cause complications recorded post-operatively were less in patients receiving iNO at both Center A and B (30 vs. 43 at Center A and 14 vs. 40 at Center B) (Tables 1 and 2).

**Table 4:** The Distribution, Frequency and Type of Hepatobiliary Complications.

	1-month post-transplant	6-months post-transplant	9-months post-transplant
**Center A**			
Placebo	5	0	0
iNO	2	0	0
**Center B**			
Placebo	3	3	3
iNO	0	0	2

Center A.

Placebo: allograft dysfunction (1), primary graft non-function (1), reduced portal vein flow (1), hepatic artery bleeding (2).

iNO: allograft dysfunction (1), hepatic artery bleeding (1).

Center B.

Placebo: 1-month:primary graft dysfunction (1), reduced hepatic artery blood flow (1), biliary leak (1), 6-months:biliary stricture (2), 9-months:rejection (1), hepatic artery stenosis (1), death (1).

iNO: 9-months:hepatic artery stenosis (1), death (1).

### Effects of iNO on liver histology, PMN accumulation and cell death

**Figure 9:**
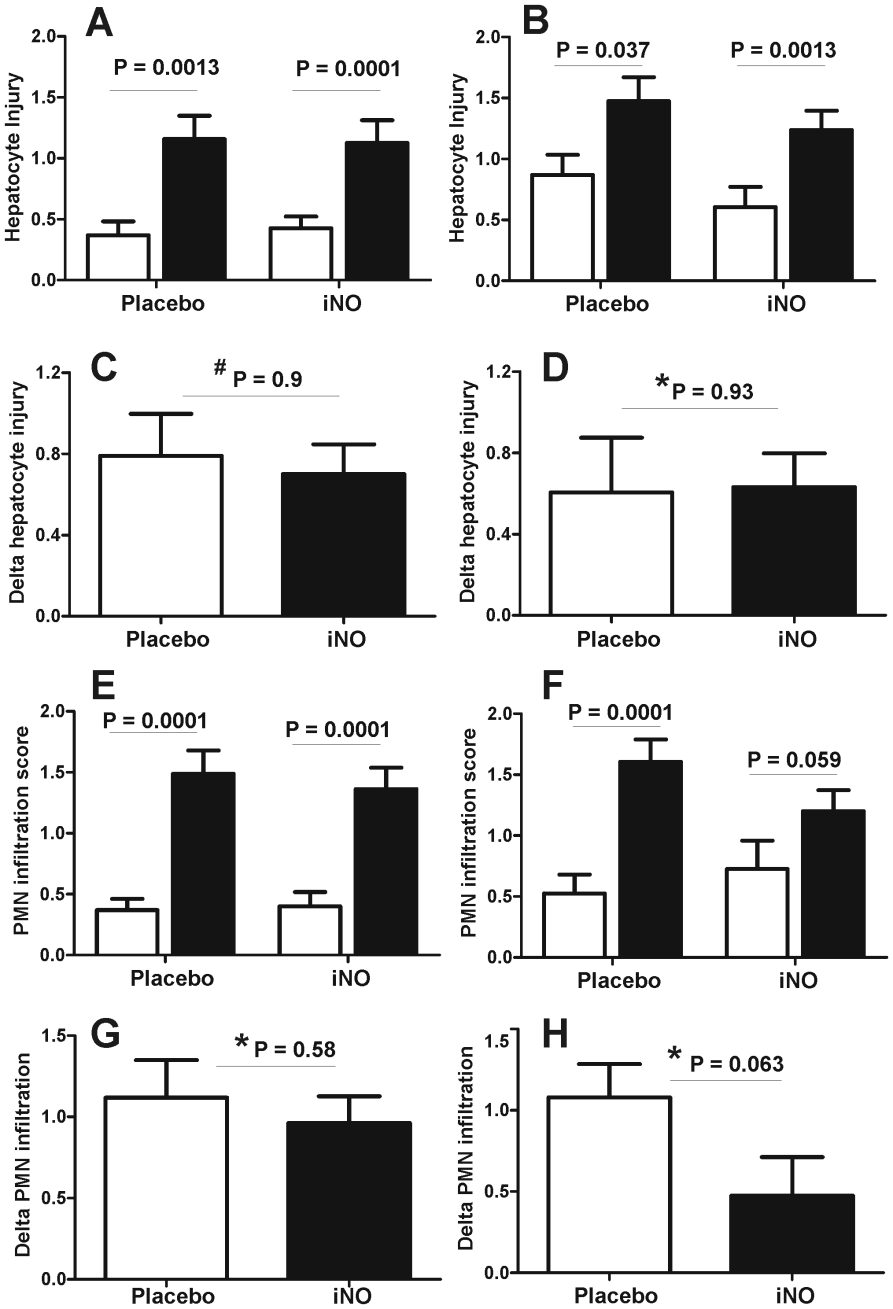
Effects of iNO on reperfusion induced injury and PMN accumulation. Liver biopsy samples were collected pre- (LB1, □) and 1 hr post dual reperfusion (LB2, ▪) and assessed for injury by histopathologic evaluation (Panel A, B) and infiltration of PMN (panel E,F). The increase (LB2 – LB1) in pathology score and PMN infiltration are shown in panels C–D (center A) and G–H (Center B), respectively. P-values indicated on graph are by paired t-test for panels A–B or by * unpaired t-test or ^#^Mann-Whitney U (panels C–D). Panels A, C, E, G from Center A cohort. Panels B ,D, F, H are from the Center B cohort.

**Figure 10:**
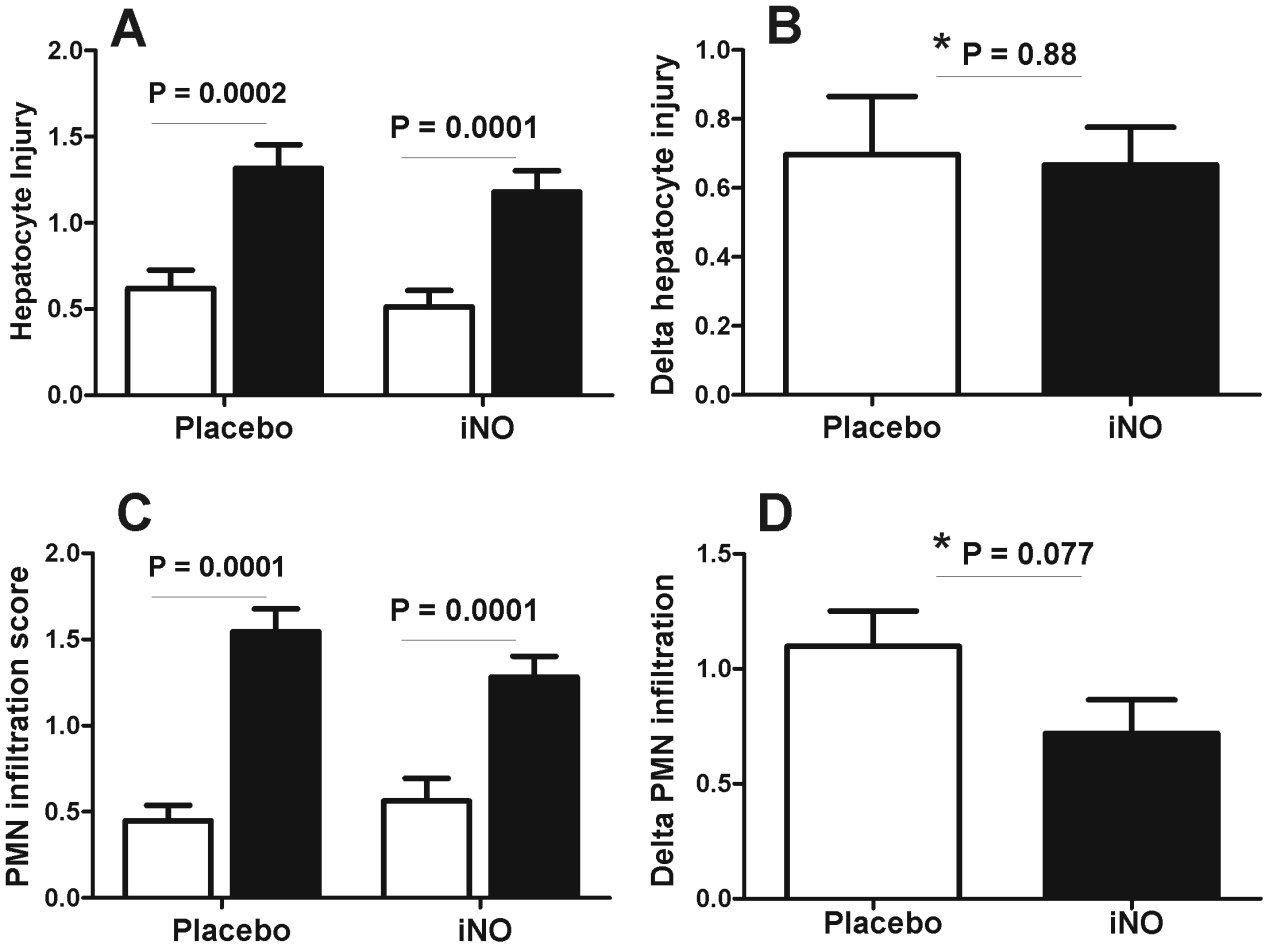
Effects of iNO on reperfusion induced injury and PMN accumulation. Liver biopsy samples were collected pre- (LB1, □) and 1 hr post dual reperfusion (LB2, ▪) and assessed for injury by histopathologic evaluation (Panel A, B) and infiltration of PMN (panel C, D). Data from both UAB and UW cohorts are combined (n = 38). P-values indicated on graph are by paired t-test for panels A and C or by * unpaired t-test (panels B and D).

**Figure 11:**
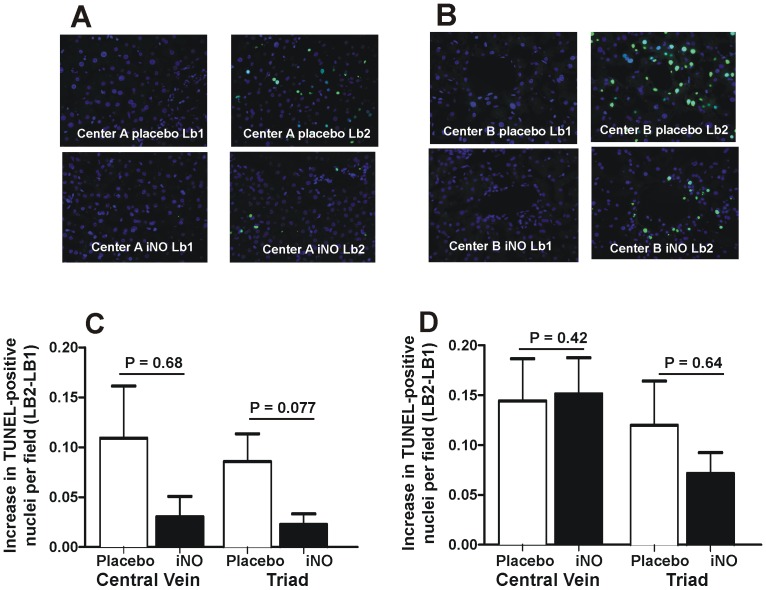
Effects of iNO on TUNEL staining pre- and post-reperfusion. Hepatic cell death was assessed by TUNEL staining in central vein and triad regions pre- (LB1) and 1 hr post-dual reperfusion (LB2) in placebo and iNO treated patients. Panel A and B show representative immunofluorescence images for TUNEL staining (green = TUNEL positive cell, blue is Hoechst 33342 staining of nuclei, magnification 40×). Panel C and D compare the magnitude of increase in TUNEL staining between placebo and iNO groups for Center A and Center B cohorts respectively. Indicated P-values calculated by Mann-Whitney U-test.

**Figure 12:**
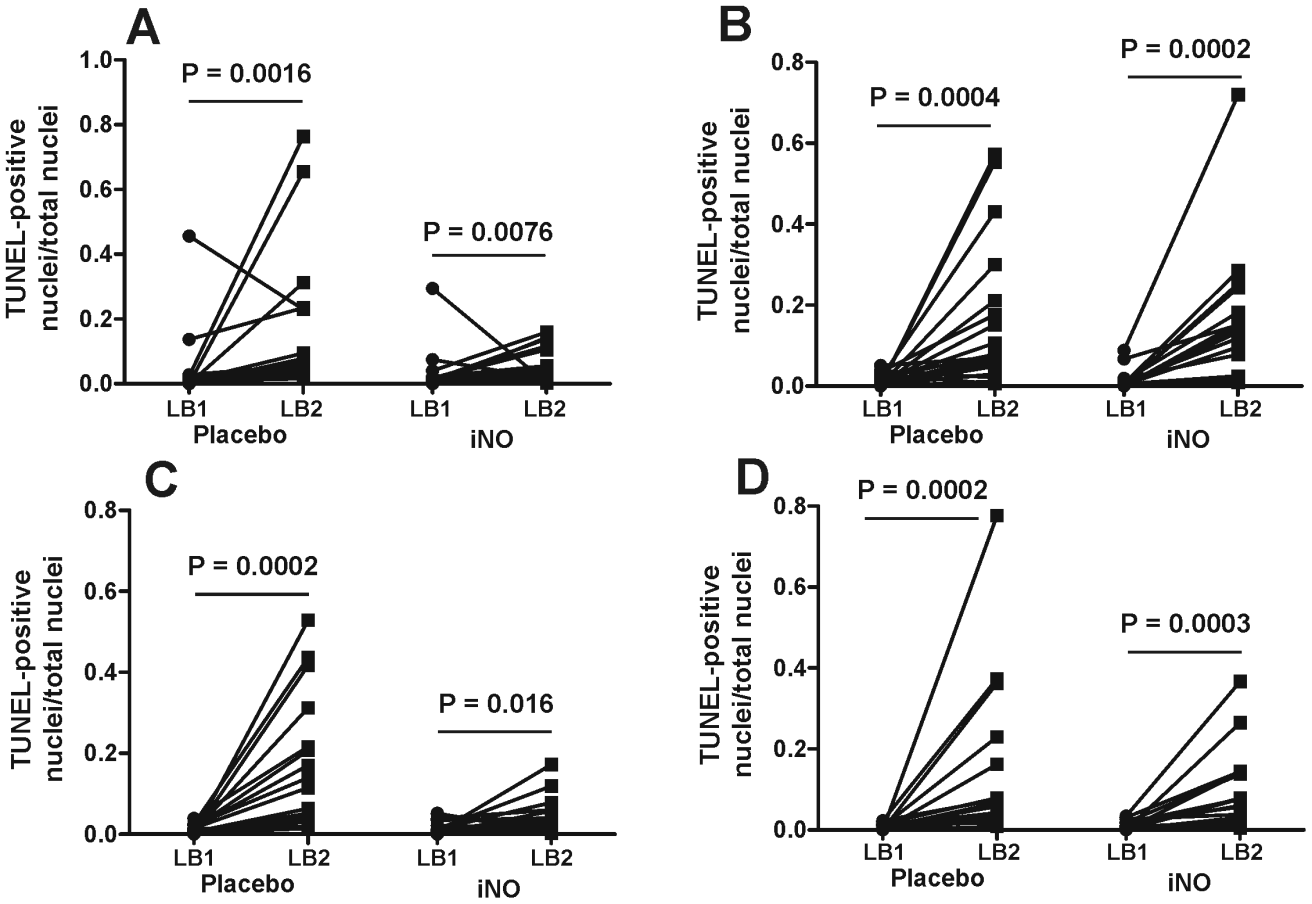
Individual responses to iNO on TUNEL staining pre- and post-reperfusion. Panel A and B show changes in TUNEL positive nuclei in hepatic central vein area and panels C and D in the hepatic triad area for Center A (panel A and B) and Center B (panel C and D) cohorts. Indicated P-values determined by Wilcoxon rank-sum (n = 18–20).

Significant increases in liver injury occurred post-reperfusion (LB2 vs. LB1) in both placebo and iNO groups at both centers. The magnitude of increase in injury (LB2-LB1) was not different between iNO and placebo in either cohort (Figure 9). Similar changes were observed for changes in PMN accumulation, although a trend towards a decrease in PMN accumulation by iNO in the Center B cohort was noted, which persisted when data from both centers were combined (Figure 10). Figure 11A–B shows representative TUNEL staining images in paired pre- and post-reperfusion biopsy samples. Figure 12 displays that in both Center A and Center B cohorts; significant increases in TUNEL staining were observed 1 hr-post dual reperfusion. Figure 11C–D show that the magnitude of this increase in TUNEL staining was not affected by iNO therapy in the Center B cohort, but trended towards being significantly diminished at Center A in the patients receiving iNO.

### Changes in NO-metabolites

**Figure 13:**
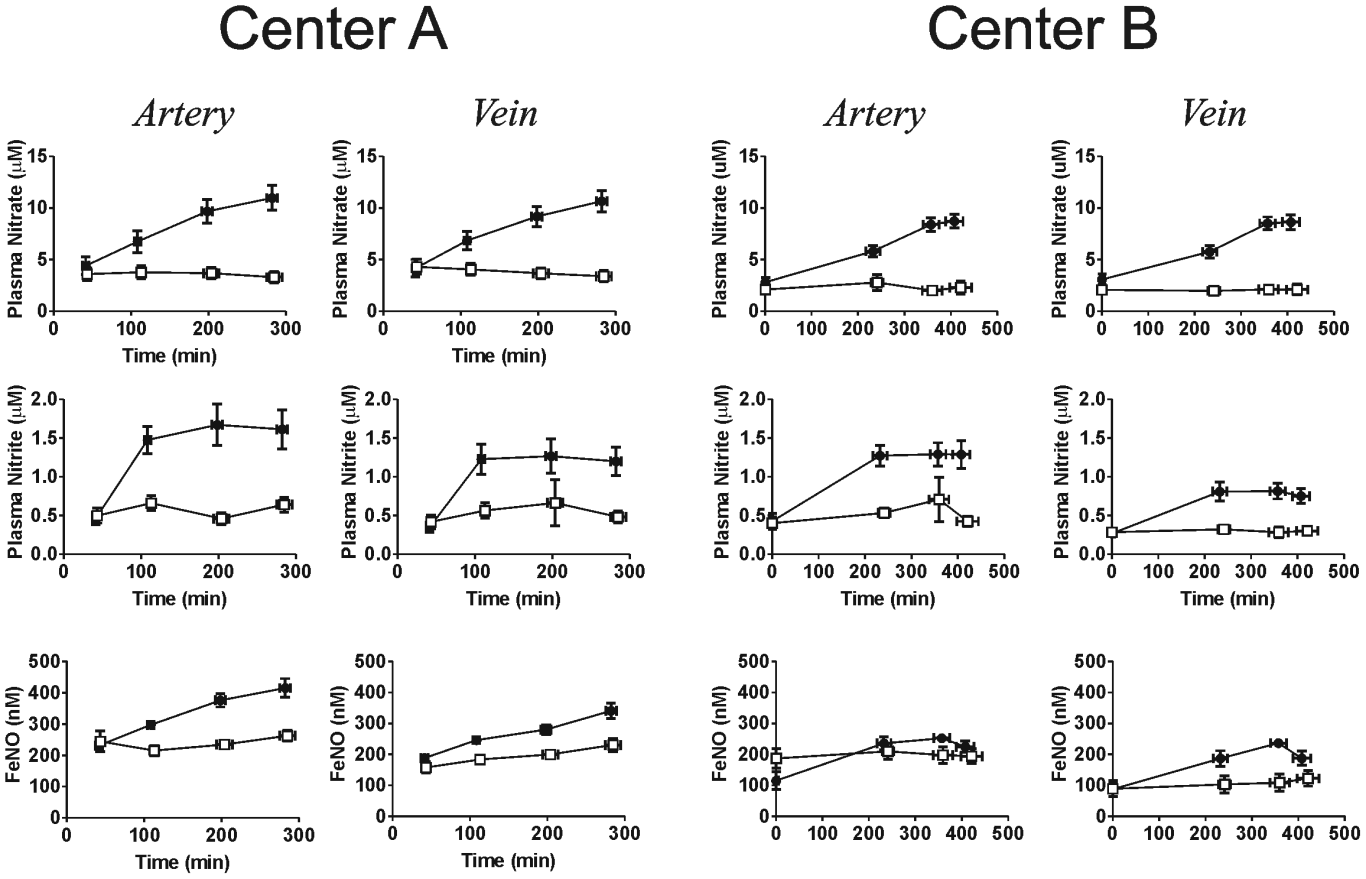
Effects of iNO on NO-derived metabolites. Plasma nitrate, nitrite and RBC FeNO (nitrosylhemoglobin) was measured in paired arterial and venous samples collected at different times during the intraoperative period (□ = placebo; • = iNO). The first time point denotes pre- initiation of placebo or iNO. The third and fourth data points represent pre- and 1 hr post-reperfusion samplings. Data show mean ± SEM (n = 19–20). P<0.001 by 2-way ANOVA for all placebo vs. iNO comparisons (except FeNO in arterial group for Center B).

**Figure 14:**
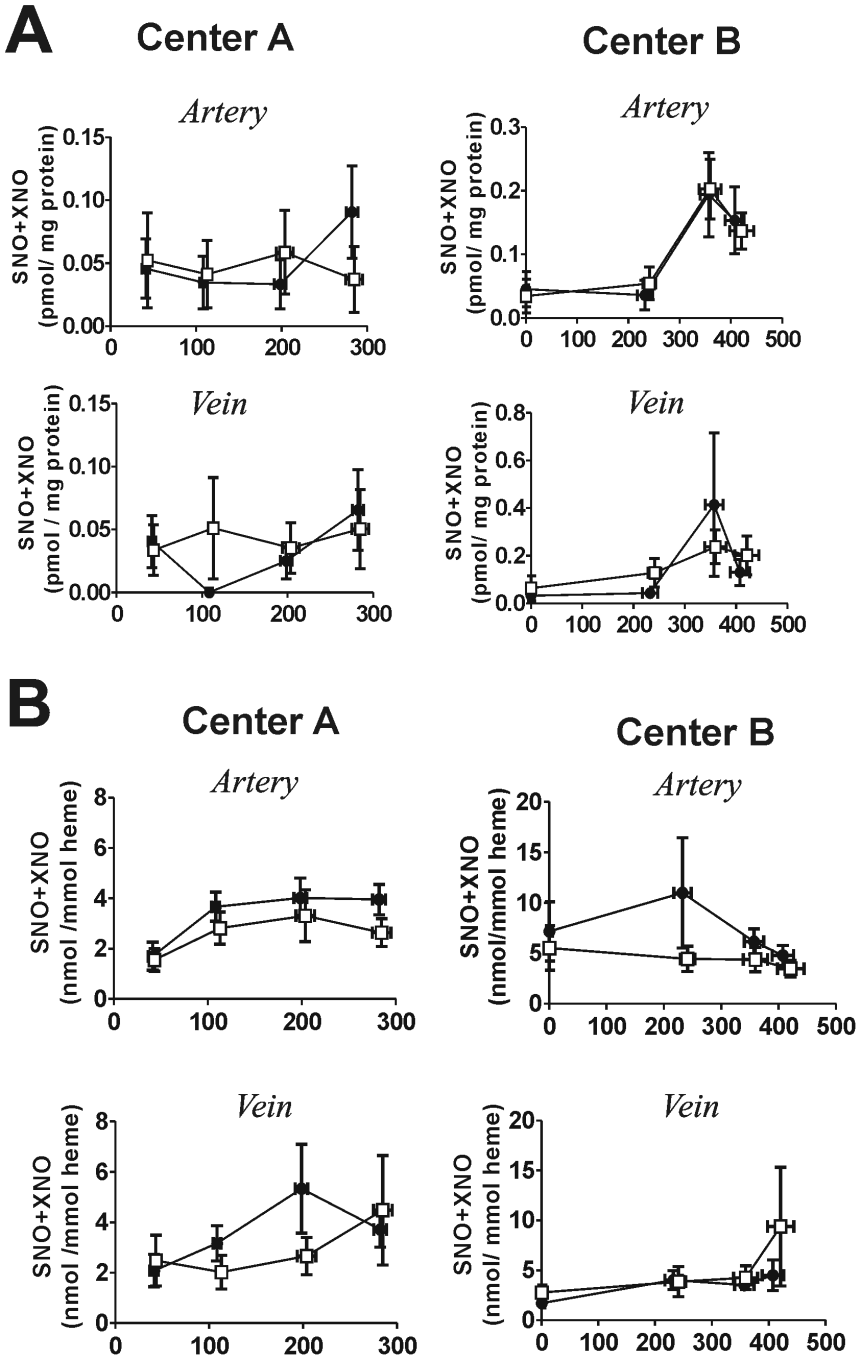
Effects of iNO on plasma and RBC nitroso species. Plasma nitroso (Panel A) or RBC nitroso (Panel B) were measured and normalized to protein and heme, respectively. Data show measurements from paired arterial and venous samples collected at different times during the intraoperative period (□ = placebo; • = iNO). The first time point denotes pre-initiation of placebo or iNO. The third and fourth data points represent pre- and 1 h post-reperfusion samplings. Data show mean ± SEM (n = 19–20).

**Figure 15:**
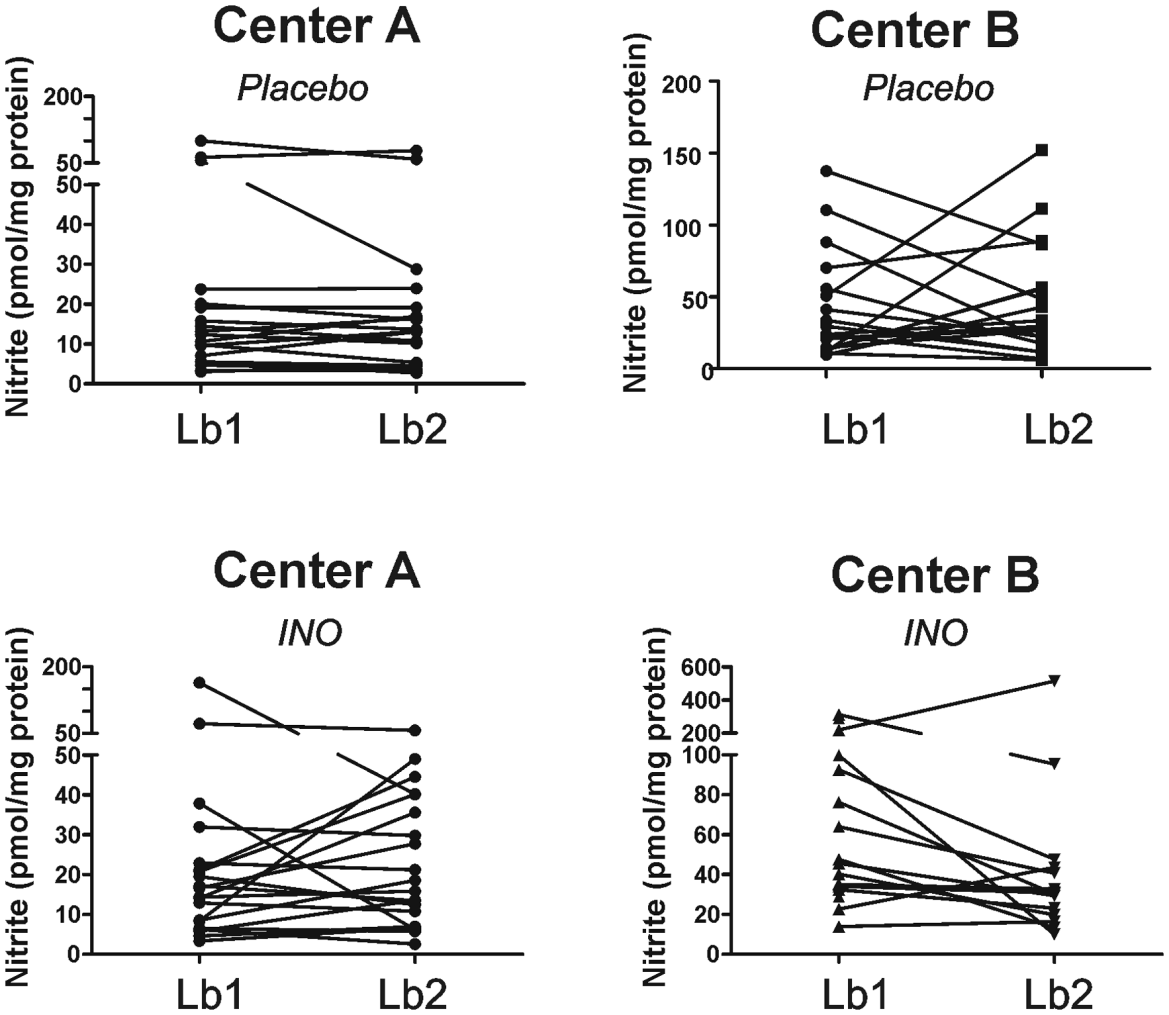
Effects of iNO on liver nitrite levels pre- (LB1) and post-reperfusion (LB2). Nitrite was measured in paired liver biopsies and data normalized to protein. No significant differences in nitrite levels were observed pre- vs. post-reperfusion or between placebo and iNO treatments.

**Figure 16:**
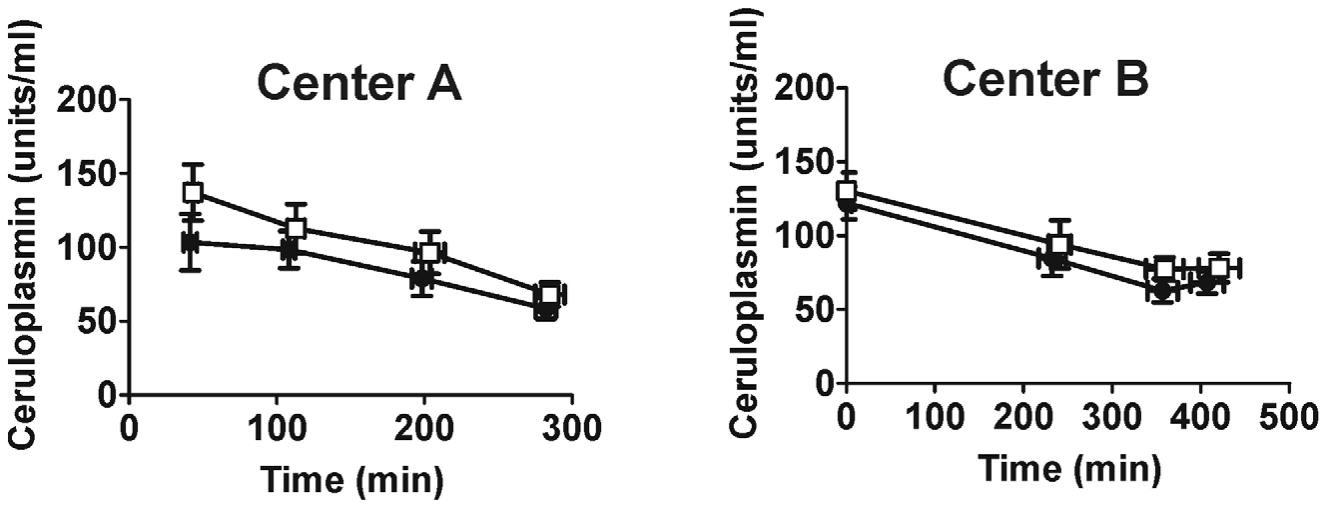
iNO effects on plasma ceruloplasmin activity. Plasma ceruloplasmin activity was measured in venous samples collected during surgery from patients administered placebo (□) or iNO (•). Data show mean ± SEM (n = 19–20).

Exposure to iNO significantly increased plasma nitrate, nitrite and RBC nitrosylhemoglobin concentrations (Figure 13), with no significant changes in neither plasma nor nitroso species evident (Figure 14). No changes in liver nitrite were observed (Figure 15). The rate of nitrate formation was faster in Center A vs. B (0.028 µM/min vs. 0.015 µM/min respectively calculated by linear regression analysis). Plasma nitrite levels reached steady state in all groups receiving iNO with arterial levels being higher relative to venous at both centers. Moreover, nitrite and nitrate levels in iNO group were higher in Center A vs. Center B cohort (P<0.01 by 2-way ANOVA). Nitrosylhemoglobin was also higher in Center A vs. Center B. Ceruloplasmin, a possible modulator of nitrite levels, decreased in both centers and all groups, with no differences between placebo and iNO, or between centers observed (Figure 16) [21].

## Discussion

Our two-center trial reveals that preemptive iNO administered to patients undergoing liver transplantation prospectively enhances allograft function and reduces complications at 9-months when assessed post-hoc. Trends towards reducing hepatocellular TUNEL positive cells and allograft PMN accumulation were also observed. Inhaled NO did not reduce ICU or hospital length of stay, nor was a definitive mechanism of hepatoprotection evident. This study is consistent with previous publications demonstrating protective effects when iNO is administered at moderate to high concentrations (20–80 ppm) prior to a predictable ischemia-reperfusion [10], [11], [16]. Together with our previous studies, administration of iNO at 80ppm was not associated with toxic accumulations of nitrogen dioxide, methemoglobin formation or bleeding complications [10], [11]. In fact, in one study, patients receiving iNO received fewer platelet transfusions compared to placebo. Thus, iNO appears to be safe in high-risk patients even if given for intervals up to 14 hrs and allays previous concerns regarding toxicities and side effects of iNO administration. [22], [23], [24], [25], [26].

In our prior study, LFTs improved significantly faster in patients treated intra-operatively with iNO at 80 ppm compared to placebo [11]. Multivariate analyses confirmed this observation when both centers were combined (“Combined” ALT group; Figure 4) but with a more profound effect at Center B. The current study corroborates findings of our earlier study but curiously is less significant at Center A where the study was originally conducted, with some end points including length of stay, RBC transfusions showing no differences between placebo and iNO in the current study, whereas they did in our earlier study. The current study protocol and demographics between Center A and the previous study did not differ except for larger enrollment with the current study. Whilst the basis of differing LFT outcomes remains unclear, we do note that AST levels measured <1 h post-surgery were ∼20–30% lower in the current study (across placebo and iNO groups) compared to our previous study suggesting that one reason for less significant effect of iNO in Center A is a lower baseline IRI. This is further suggested by more significant effects of iNO in Center B vs Center A in the present study (see below).

Effects of iNO on histologic injury were not significantly different between the iNO and placebo groups at either center. The degree of injury assessed by histology observed at Center A between the iNO and placebo groups were essentially identical. Inhaled NO has been previously demonstrated to possess an anti-PMN effect via reduced levels of endothelial cell adhesion molecules [10], [14], [27], [28]. These findings were seen in our initial human study investigating iNO's efficacy in patients undergoing tourniquet-induced lower extremity IRI. However, in our subsequent study involving patients undergoing liver transplantation, a definitive anti-neutrophil effect due to iNO was not seen. The current trial delivered similar results with Center A being consistent with our previous findings in patients undergoing liver transplantation. However, iNO delivered to patients at Center B attenuated PMN accumulation more so than Center A.

Previous literature supports preemptive or early use of NO in modulating apoptosis and necrosis [6], [29], [30], [31]. Decreased TUNEL staining trended towards significance in the iNO group at Center A, consistent with previous findings. However, iNO did not significantly affect TUNEL staining in Center B. Collectively, these data demonstrate that assessing IRI in liver transplantation by clinical LFTs is not necessarily correlative with histologic or biochemical markers of injury, and underscores the fact that IRI is complex with no one functional outcome being sufficient for evaluation of severity. That said, iNO therapy did improve allograft function per LFTs, but with a stronger effect at Center B. We postulate that more IRI was likely encountered in the Center B cohort, secondary to longer surgery times, higher MELD and WIT and that these differences may underlie the more profound effect of iNO in this group. This is also suggested by ∼2-fold higher AST levels post-surgery in Center B vs Center A cohorts (see Figure 6 legend). Differences in the severity of IRI may also have contributed to lesser effect of iNO observed in Center A, compared to our previous study performed using the same protocol and at the same institution (see above). Other recent publications utilizing models of non-heart beating donors and transplanted steatotic livers also support NO in significantly reducing ALT levels, enhancing microcirculatory perfusion and reducing other injury indices [32] [33]. A clear limitation in this conclusion and study design was that assessment of IRI only occurred 1 hr post-reperfusion in both centers. Differences in the sensitivity of the allograft to undergo IRI is likely to be reflected in kinetics of this process and may underlie why differences in iNO effects on biochemical and histologic parameters between Center A and Center B were observed.

How iNO mediates extra-pulmonary effects remains unclear with the general hypothesis being that iNO forms a relatively stable, NO-containing intermediate in the circulation, which then mediates systemic effects either directly or after being recycled to NO [34]. Evidence in a feline model of IRI suggested the intermediate may be plasma S-nitrosothiols (e.g. S-nitrosoalbumin), whereas studies in humans and mice indicate nitrite as a possible mediator [35]. A key role for nitrite is also indicated by its direct administration protecting against hepatic IRI in murine models [36] and the description of biological mechanisms for nitrite reduction to NO under ischemic conditions. It should be noted that other NO-containing candidates in the circulation that are relatively labile under biological conditions and may also be formed upon the inhalation of NO (via nitrosylation or S-nitrosation reactions). These include S-nitrosothiols in the RBC, ferrous-nitrosylhemoglobin (HbNO) and C- or N-nitrosamines (referred to as XNO) [34], [37], [38], [39]. Similar to our previous results, iNO increased only circulating nitrate, nitrite and nitrosylhemoglobin. Several studies have demonstrated therapeutic nitrite administration can prevent IRI in multiple organ systems and thus represents a likely candidate for transducing extra-pulmonary effects of iNO [40]. Interestingly, nitrite levels reach steady state with nitrate and nitrosylhemoglobin increasing continuously during iNO administration. This is consistent with known chemistry of nitrite reactions namely oxidation by oxyhemoglobin to nitrate and reduction by deoxyhemoglobin (or other metalloproteins) to NO, which together with direct binding of iNO with any deoxyhemoglobin in the pulmonary circuit, likely leads to the observed increase in nitrosylhemoglobin. However, all iNO-dependent increases in NOx were higher in the Center A cohort compared to Center B. This was a surprising result and suggests center/demographic specificity with respect to how iNO is metabolized. We also acknowledge the limitation that nitrite levels only reached ∼1.5 µM steady state; which may not be optimal for therapeutic benefit; a question that could be tested by using reagent nitrite based therapy.

Of interest were the reduced complication rates at 9-months post-transplantation that we termed “hepatobiliary specific”. These included primary non-function, hepatic artery and portal vein stenosis, allograft rejection and bile duct stenosis requiring stent placement. Patients receiving iNO experienced a smaller number of these complications compared to placebo. This data was gleaned from a post-hoc analysis. We appreciate the limitations of these analyses and note that the study was not likely powered for this outcome and that there are many factors independent of iNO that could contribute to different rates of hepatobiliary complications including surgical technique and surgeon heterogeneity. With these limitations noted, we feel it is important to report these findings for consideration for future studies, and note that there are plausible mechanisms by which iNO could have been beneficial including improved hepatic and biliary microcirculatory flow, less hepatocellular necroapoptosis and thrombosis at the site of vascular endothelial injury. Whilst we cannot account for all these, the timing of the observed complications occurred with equal frequency with half occurring within the two weeks and others between 3–9 months postoperatively. Longer term effects affording protection might include diminishing early inflammatory effects of IRI reducing concentrations of reactive oxygen species, reduced adhesion of sinusoidal neutrophils, reduced cytokines and reduced dendritic cell recruitment. Reduction in dendritic cell migration and recruitment due to NO has been demonstrated [41]. Dendritic cell reduction may serve to diminish the number of activated naïve alloreactive T cells, thus reduce the risk of allotransplant rejection. Lastly, it is known that patients receiving livers from non-heart beating donors have a higher probability of ischemic- induced cholangiopathy [42]. In this trial, 2 patients received livers from non-heart beating donors. Both were from Center B and both were in the placebo limb. One patient did develop a biliary stricture requiring biliary stent placement. However, overall the biliary injury rate was below the expected mean at both centers [41]. Interestingly, animals subjected to cardiac arrest and administered L-arginine, a precursor of NO, had significant reductions in ischemic cholangiopathy [43].

Definitive conclusions regarding the use of iNO as a preemptive strategy to attenuate IRI in liver transplantation are not possible and the limitation of the relatively low sample size in this study has been noted. However, an important consideration is that that higher complication rates result in a higher readmission rates and therefore higher costs per patient transplanted. Cost to administer iNO at present day non-contract rates are $163/hr. However, these costs would appear fairly trivial within the context of the costs of treating patients undergoing liver transplantation. The average costs for continuous intraoperative iNO administration were $750 for Center A and $1,290 for Center B, respectively. Taking into consideration that iNO therapy decreased post-operative hepatobiliary complications, we feel that further studies are warranted at evaluating whether iNO use should be routine in liver transplantation especially in patients where the risk of significant IRI is greater (e.g. high DRI allografts, extended criteria and DCD donors).

In summary, preemptive iNO was found to be safe, well-tolerated and efficacious to the liver allograft. Hepatobiliary-specific and overall complications were reduced when iNO was administered. Administration of iNO did not change ICU or overall hospital length of stay, but did reduce hospital readmission rates. Overall, costs of administration appear trivial when total costs for caring for these patients is considered.

## Materials and Methods

Study design, data analysis and presentation were performed solely by the investigators and were approved by the University of Alabama's Institutional Review Board for Human Use and the University of Washington's Institutional Review Board, Division of Human Subjects. The overall study protocol was similar to that as previously described [11]. Written informed consent was obtained from all participants involved in our study. The trial was registered on *ClinicalTrials.gov* with registry number 00582010 and the following URL:http://clinicaltrials.gov/show/NCT00582010. The protocol for this trial and supporting CONSORT checklist are available as supporting information; see Checklist S1 and Protocol S1.

### Patient enrollment and study design

Fifty seven patients admitted consecutively to the University Hospital, University of Alabama at Birmingham for liver transplantation were screened for participation in the study between April, 2008 and February, 2009 (Figure 1). Of these, 40 were enrolled. Patients were excluded if consent was denied (n = 1), if under 19 yrs of age (n = 4), diagnosed with hepatopulmonary syndrome (n = 4), or allograft being used for split liver transplantation (n = 1). An additional 7 patients were screened but not enrolled due to logistical constraints related to collecting samples at the bed side. One patient died in the Center A cohort during surgery from cardiac arrest. Upon unblinding, this patient was in the placebo group, and clinical review suggesting death was unrelated to study design. In parallel, 375 patients were assessed for eligibility at the University Hospital, University of Washington in Seattle for liver transplantation between May, 2009 and April, 2011 (Figure 2). Of those assessed, 335 were excluded for a variety of reasons including significant non-cardiopulmonary comorbidities (n = 66), refusing enrollment (n = 44), competing research protocols (n = 40) and significant language barriers (n = 32). An additional 5 patients died while undergoing workup for transplantation. Since IRB-directed enrollment was significantly different between the two centers, randomization was performed per center and not centrally.

Patients at each center were randomly assigned to receive either placebo (nitrogen) or iNO (80ppm). Tanks containing NO or N_2_ (placebo) gas were blinded and randomly numbered from 1–40 at each center using an internet-based random number generator similar to previously described [11]. Subsequently, sealed envelopes containing these numbers were prepared and randomly selected (upon receipt of patient consent) again using an internet-based random number generator. Assignment of placebo or iNO was blinded to study investigators and all protocols were approved by institutional IRB. Study protocol was as described previously [16]. Upon achieving a stable hemodynamic status after the induction of anesthesia, blood draw 1 (BD1) was taken and followed by the initiation of either the placebo or iNO gas, with it being continued until 1 hr post-reperfusion. Three subsequent blood draws were taken, BD2 taken ∼1 hr after BD1, BD3 immediately before portal vein reperfusion and BD4 1 hr post-dual hepatic artery and portal vein reperfusion. Wedge biopsies from the right lobe of the donor liver were collected pre-reperfusion (LB1) and 1 hr post-dual reperfusion (LB2). Clinical assessment of liver function post transplantation were evaluated by measuring serum levels of alanine transaminase (ALT), aspartate aminotransferase (AST), alkaline phosphatase (AP), bilirubin, and coagulation times, specifically prothrombin time (PT) and partial thromboplastin time (PTT). Due to significant differences in surgery times between Center A and Center B, data for blood NO metabolite levels are plotted against the surgery time, with time zero determined from surgical records.

### Blood collection and processing

Paired arterial and venous samples were collected at each blood draw. For NO derivative (NOx) measurements, samples were processed at the bedside to stabilize plasma and RBC NOx. The different NOx measures included nitrate, nitrite, nitroso species (*S*-nitroso and C/N-nitroso species) and nitrosylhemoglobin (FeNO) by HPLC-coupled to the Griess reaction or NO-chemiluminesence as previously described [11], [44], [45], [46]. Limits of detection in RBC or plasma were 100 nM for nitrate and 25 nM for other NOx. Venous plasma was also collected for measurement of ceruloplasmin activity.

### Liver biopsy processing

Upon retrieval, liver biopsies were immediately divided into equal sections and either fixed in formalin, snap frozen in liquid N_2_ or processed for nitrite measurements. Formalin fixed samples were sectioned (5 µm) and used for histochemical evaluation of hepatocellular injury, and TUNEL staining as previously described [11].

### Liver function tests

Liver function test data were collected using standard of care procedures. There were no differences in laboratory unit differences in how these tests were presented between centers.

### Histopathology scoring

Scoring was performed as previously described [11] and according to the following scoring criteria (0 - no hepatocellular damage, 1 - mild injury characterized by cytoplasmic vacuolization and focal nuclear pyknosis, 2 - moderate injury with dilated sinusoids, cytosolic vacuolization, and blurring of intercellular borders, 3 - moderate to severe injury with coagulative necrosis, abundant sinusoidal dilation, RBC extravasation into hepatic chords, and hypereosinophilia and margination of neutrophils, 4 - severe necrosis with loss of hepatic architecture, disintegration of hepatic chords, hemorrhage, and neutrophil infiltration). Criteria to specifically evaluate PMN infiltration (0 - zero, 1 - minimal, 2 - mild, 3 - moderate, 4 - severe) were also utilized.

### Assessment of postoperative hepatobiliary complications and all-cause complications

Complications relating specifically to the transplanted allograft referred to as “hepatobiliary” were recorded up to 9-months post-transplantation at both centers. This included complications such as but not limited to primary non-function, acute/chronic graft dysfunction, rejection, hepatic artery stenosis and biliary stricture. In addition, all post-operative complications occurring during the patient's initial hospitalization following transplantation were reported.

## Statistical Analysis

Forty patients per treatment group (n = 40/treatment) were proposed based on a power analysis to detect a 30% difference in complication rates with a power of 0.8 and significance level of 0.05. For biochemical measurements, repeated ANOVA with Bonferroni post-tests were used to assess time-dependent changes and differences between iNO and placebo treatment groups at each time point.

Initial evaluation (Table 1 and 2) indicated significant differences in patient and surgery demographics between Center A and Center B. Moreover, mixed modeling analysis (using LFT as primary end points) using weight, MELD, warm ischemic time (WIT), surgical times and donor related index (DRI) as covariates indicated that effect of treatment was center-dependent for select LFTs. Therefore the following analyses were performed as a combined cohort (both centers) and per center and where stated. The distributions of all quantitative measured parameters in patients receiving either placebo or iNO were first examined for normality (D'Agostino and Pearson Omnibus normality test). Differences in treatment groups were then evaluated by either the Mann-Whitney U test or by the independent samples t-test, as appropriate. Categorical measures were examined with Fisher's exact test. Complications were examined with logistic regression. Post-surgical NOx levels were recorded at multiple time points and examined with repeated measures ANOVA. Time in hospital was modeled with Kaplan-Meier and Cox-proportional hazards regression; subjects were analyzed per protocol adjusted for gender, MELD, surgery time, cold ischemic time, warm ischemic time (WIT, time between out of cooler to portal vein and hepatic artery reperfusion) (above or below 90 min), weight, DRI, BMI (above or below 25) [19] and based upon *a priori* clinical advice. All quantitative values are presented as mean ± SEM and categorical measures are presented as counts. Analysis was performed using GraphPad software and SAS version 9.2 (SAS Institute Inc, Cary, NC).

## Supporting Information

Protocol S1 (DOCX)

Checklist S1 (DOC)
