# Supplementary material for: A Randomized Clinical Trial Testing the Anti-Inflammatory Effects of Preemptive Inhaled Nitric Oxide in Human Liver Transplantation
Source: PLoS One. 2014 Feb 12;9(2):e86053. doi: 10.1371/journal.pone.0086053 (PMC3922702; doi:10.1371/journal.pone.0086053)
Supplement: Protocol S1 — (DOCX) [file pone.0086053.s001.docx]

**Clinical Protocol (ClinicalTrials.gov Identifier:NCT01172691)**

Hepatic ischemia-reperfusion (no blood flow and then reestablishing blood) leads to significant systemic inflammatory response (an infection-like response). This response involves numerous complex reactions that ultimately contribute to liver injury. The consequences of these actions may lead to acute graft dysfunction (the new donor liver to malfunction at the time of or shortly after surgery)/ failure or transient graft dysfunction (temporary malfunction of the new donor liver), but may also be responsible for other organs to fail, such as lung and kidney. Recent studies in children and adults document the effectiveness with inhaled NO. Inhaled NO has shown effectiveness in improving a number of clinical syndromes associated with high pressures in the lungs such as: (1) adult respiratory syndrome, (2) chronic obstructive pulmonary disease, (3) mitral valve disease, (4) cardiac and lung transplantation, (5) persistent pulmonary hypertension of the newborn, and (6) congenital heart disease. Surprisingly, the potential anti-inflammatory (decreased inflammation) properties of administering NO before the liver is transplanted has remained unexplored clinically, while a considerable number of well-designed liver surgery animal studies have demonstrated benefit. We recently demonstrated that iNO administered during liver transplantation enhanced allograft function, reduced transfusion requirements and decreased overall hospital length of stay. We now propose an additional 2-center prospective randomized clinical trial to test the hypothesis that iNO will enhance allograft function (primary endpoint) and reduce ICU/hospital length of stay (secondary endpoint).

This study will be conducted at the University of Washington Medical Center and the University of Alabama at Birmingham. The population from which derived are patients aged 19 years and older who are scheduled to undergo liver transplantation. Approximately 40 participants (those that will receive nitric oxide) and 40 controls (those that will not receive nitric oxide) will be enrolled. The 40 controls would be cared for per standard of care. Meaning, no iNO will be delivered but blood and liver specimens will be obtained per the protocol. We will obtain consent from all participants including the controls. Participants will be randomly assigned (like flipping a coin) to either receive nitric oxide or standard of care. This will be achieved by selecting blinded envelopes containing the assignment group.

The past experimental and clinical findings lead to the formulation of this study. Analysis of 80 patients randomly assigned inhaled nitric oxide (n=40) or control (n=40) reveal no adverse effects in treatment group. Moreover, patients receiving nitric oxide show trends towards lower hospital length of stays and hepatic ischemic reperfusion injury. Experimental data suggest that nitric oxide metabolites (chemicals produced as a result reactions with NO) in the circulation increase in the nitric oxide group compared to control. The current protocol builds upon preliminary findings discussed above together with the literature and the PI’s knowledge and expertise in nitric oxide clinical research.

Patient enrollment: Patients advised of the nature and risks of the study, which are willing to give informed consent, will be enrolled in the study. If a patient is unable to give informed consent, approval will be obtained from next of kin. The patient will undergo induction of anesthesia adhering to standard protocol. Inhaled NO will be initiated 30 minutes after the induction of anesthesia and continuously delivered at 80 ppm until the case is completed (Figure A.). Continuous measurements of nitrogen dioxide and intermittent measurements of methemoglobin will be obtained to ensure no toxicities are encountered. Blood samples to help explain the effect of iNO will be obtained 30 minutes after induction of anesthesia, during the anhepatic phase (time when there is no liver in the patient), and 60 minutes post-hepatic reperfusion (time when both the significant blood vessels have been sewn together and are providing blood to liver). Liver function test data will be collected using standard of care procedures.. Sreum samples will also be analyzed for serum nitric oxide metabolites, markers of nitric oxide reactivity, markers of liver injury, inflammatory markers and cytokines. Just prior to and after reperfusion, liver biopsies (core or wedge-at the discretion of the surgeon) will be performed on the right lobe and immediately processed for subsequent analysis. Liver biopsy specimens will be analyzed for the following: (1) histological analysis with hematoxylin and eosin staining will be performed on 3 µm sections on liver biopsy tissue obtained pre and post-reperfusion. (2) The liver biopsy specimens will assessed for hepatocellular necroapoptosis using the TUNEL method and for measurements of caspase-3. Complications will be recorded for up to 9-months post-transplantation at both centers. The 40 controls would be cared for per standard of care. Meaning, no iNO will be delivered but serum and liver specimens (core or wedge-at the discretion of the surgeon) will be obtained per the protocol. Pertinent clinical data will be tracked post-operatively for up to one year to assess for post-operative morbidity & mortality and to assess the potential effect NO on immunosuppressive regimens and doses of the particular immunosuppressive agents. We will obtain consent from all participants including the controls. Participants will be randomly assigned to either receive nitric oxide or standard of care. This will be achieved by selecting blinded envelopes containing the assignment group.

**Figure A. iNO Therapy and human liver transplantation**

(A) Experimental Protocol for administering Placebo or iNO to patients and sample (blood and liver biopsy) collection. (BD = blood draw).

Since Participants will be randomly assigned to either receive nitric oxide or standard of care by selecting blinded envelopes containing the assignment group, the participants’ group assignment will not be released during the study. The PI and co-investigators will disclose the assignment information and research results to participants at the end of the study.

The study will include male/female, all races and ethnic composition. During the study period, any critically ill patients aged 19 years and older who are scheduled to undergo liver transplantation will go through the screening procedures.

Inclusion Criteria: Males or non-pregnant females at the University of Washington and the University of Alabama at Birmingham undergoing orthotopic liver transplantation who, in the opinion of the attending physician are likely to acquire hepatic injury during reperfusion.

Exclusion criteria: (1) Patients with a known history of an adverse reaction or toxicity sensitivity to NO, (2) Inability to safely deliver NO for any reason, (3) Hepatopulmonary syndrome.

Patients will be recruited from the operative schedule of surgeons. Each patient and/or significant other will be provided with a copy of the completed, signed consent form. The investigators or their representatives will explain the potential risk, discomfort, benefits and the right that subject may choose not participate in the study, and that subjects are free to withdraw their consent and not to participate in this project without prejudice against further care that they may receive at this institution.

The study procedures will be carried out in Operating Room (OR) of University of Washington Medical Center and the University of Alabama at Birmingham.

All patients will be exposed to the inherent risks of their underlying disease. Potential adverse effects of inhaled NO therapy include: (1) airway cytotoxicity in susceptible patients due to over production of and/or impaired defenses against toxic NO metabolites such as nitrogen dioxide (NO2), (2) excessive production of methemoglobin during NO administration ,and (3) NO-mediated decreases in platelet adhesiveness and aggregation. To date, available information suggests a low, dose- related and reversible incidence of these adverse effects in patients receiving NO.

Extensive, continuous, physiologic monitoring will be used in all patients to detect hemodynamic and metabolic abnormalities. Inspired concentrations of NO and NO_2_ will be continuously monitored with an extremely sensitive chemiluminescent or electrochemical analyzer. Methemoglobin levels and arterial blood gas sampling will be performed on induction, and generally every 60 minutes from that point on and or as necessary per the patient’s clinical course. In detail, the following hazard avoidance procedures will be used: (1) Minimal effective doses of therapeutic agents will be carefully titrated using precise delivery systems, (2) Inspired concentrations of NO and NO_2_ will not exceed known toxic levels of 3 ppm, (3) Ambient concentrations of NO and NO_2_ will be monitored and expired gases scavenged as necessary to prevent environmental pollution and (4) methemoglobin concentrations exceed 5%. An individual subject would be terminated from the study if inhaled nitric Oxide exceeds concentrations of > 3 ppm for > 1 hour. Concentrations of NO_2_ and indices of oxygenation will continued to be monitored throughout the intra and postoperative course to ensure patient safety.

The information gained in this study will allow clinicians greater knowledge of potential benefits of inhaled nitric oxide and the prevention of liver injury. Such information may assist in developing better treatment for prevention of liver injury associated with liver transplantation. Current information suggests that the overall risk from selective pulmonary vasodilatation with inhaled NO is extremely small. Potential toxicities (i.e., methemoglobin formation and NO2) are being monitored so adjustments in delivered NO concentrations can be easily made. The potential alteration in platelet adhesion and aggregation has been demonstrated to be clinically relevant.

Patient specific information collected during this study will be kept confidential in a locked, secure location. Only the investigators and staff directly involved with the study will have data access. The information gathered during this study will be kept confidential to the extent permitted by law. However, subject’s doctor, representatives of NIH, the U.S. Food and Drug Administration (FDA) and University of Washington's Institutional Review Board (IRB) will be able to inspect his or her medical records and have access to confidential information. The results of the study may be published for scientific purposes; however, subject’s identity will not be revealed.

The specimens will be anonymous using the numerical system like a numerical sequence i.e. 101, 102. All specimens shared with other investigators will be stripped of all identifiers except anonymous numerical code assigned as above. Prior to sharing specimens, documented IRB approval from recipient investigator for use of specimens will be required.

For screening purpose, patients’ medical records will be reviewed to check their history of following aspects, (1) Patients with a known sensitivity to NO, (2) Inability to safely deliver NO for any reason.
